# Ruptured Appendiceal Mucocele Presenting as a Ventral Hernia: A Case Report of a Rare Presentation of Appendiceal Mucocele

**DOI:** 10.7759/cureus.23304

**Published:** 2022-03-18

**Authors:** Emtenen M Meer, Abdullah R Khazindar

**Affiliations:** 1 Radiology, King Faisal Specialist Hospital and Research Centre, Jeddah, SAU; 2 Department of Radiology, College of Medicine, University of Jeddah, Jeddah, SAU

**Keywords:** pseudomyxoma peritonei, abdominal mass, abdominal hernia, right adnexal lesion, mucocele of the appendix, right lower quadrant pain

## Abstract

Mucocele of the appendix (MA) is a rare disease characterized by chronic accumulation of mucin within an appendix. Although MA can be an asymptomatic finding, some patients with MA may present with right lower quadrant (RLQ) pain, pelvic pain, or even hernias. The later presentation is usually related to rupture of the mucin-filled appendix, a condition referred to as pseudomyxoma peritonei (PMP). Herein, we present a case of ruptured MA presenting as an irreducible paraumbilical hernia, where the patient presented with a lump to the surgical clinic. computed tomography (CT) of the abdomen revealed an RLQ lesion extending through the hernial neck. Further characterization of the lesion was performed with magnetic resonance imaging (MRI), revealing an appendiceal origin of the lesion. The patient underwent an exploratory laparotomy during which an omental sample was taken. Histopathology confirmed the diagnosis of metastasizing low-grade appendiceal mucinous neoplasm. We believe that our case is unique due to the rarity of ruptured MA as well as the rarity of it presenting as a paraumbilical hernia.

## Introduction

Mucocele of the appendix (MA) was first described by Rokitansky in 1842 and defined as appendiceal lumen dilatation due to accumulation of mucus [1]. Although it is a rare disease that accounts for 0.2-0.7% of appendiceal pathology, it is very important to know and to consider in the differential diagnosis [2]. Clinical presentation of MA is variable; half of the patients will be asymptomatic while others might present with abdominal pain, acute appendicitis, right lower quadrant (RLQ) mass as in our case or even with intestinal strangulations [3]. MA could be benign or malignant. Although it can be seen by ultrasonography (US), it is commonly identified utilizing CT scan. The most common feared complication is rupture of the mucocele, which leads to pseudomyxoma peritonei (PMP). The treatment is mostly appendectomy, however, if malignancy is suspected a right hemicolectomy should be performed.

## Case presentation

**Figure 1:**
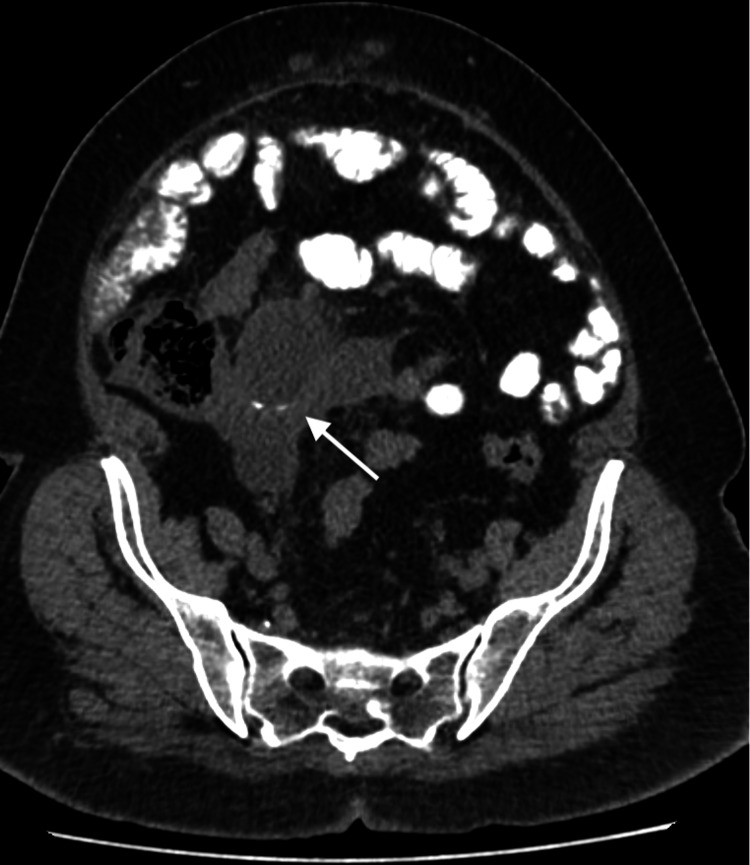
Axial CT imaging of the lower abdomen with administration of oral contrast. Axial view of the lower abdomen demonstrates a large irregular hypodense mass in the right lower quadrant inseparable from the cecal pole. A rounded, well circumscribed cyst is seen within the mass and is associated with a curvilinear calcification on its wall.

**Figure 2:**
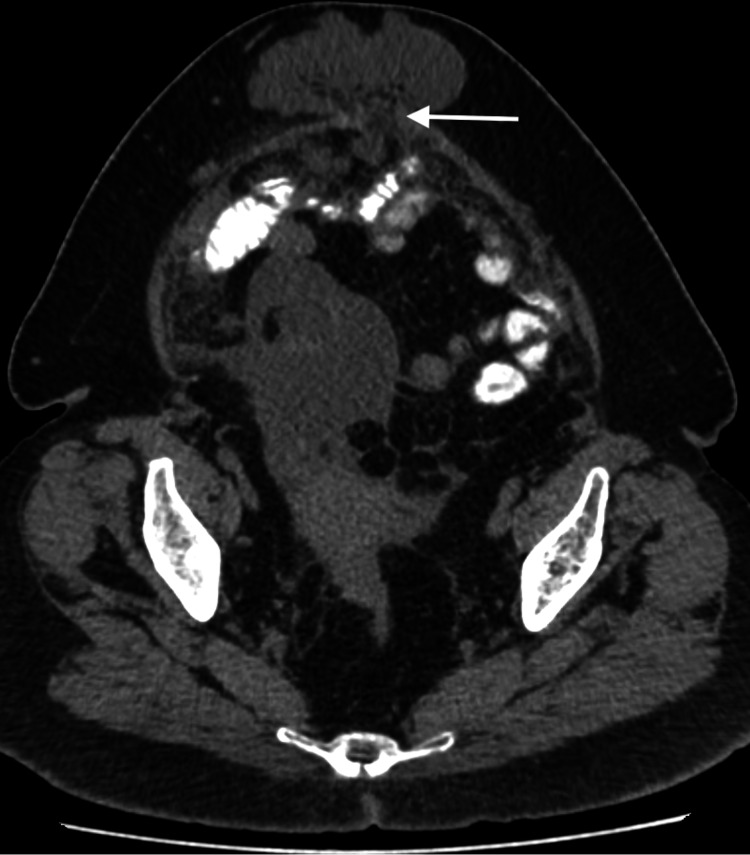
Axial CT imaging of the lower abdomen with oral contrast. Axial view of the lower abdomen demonstrates extension of the mass from the right lower quadrant to the anterior abdomen. The mass is seen herniating through a defect in the anterior abdominal wall (arrow). Fat stranding is also seen at the neck of the hernial sac.

**Figure 3:**
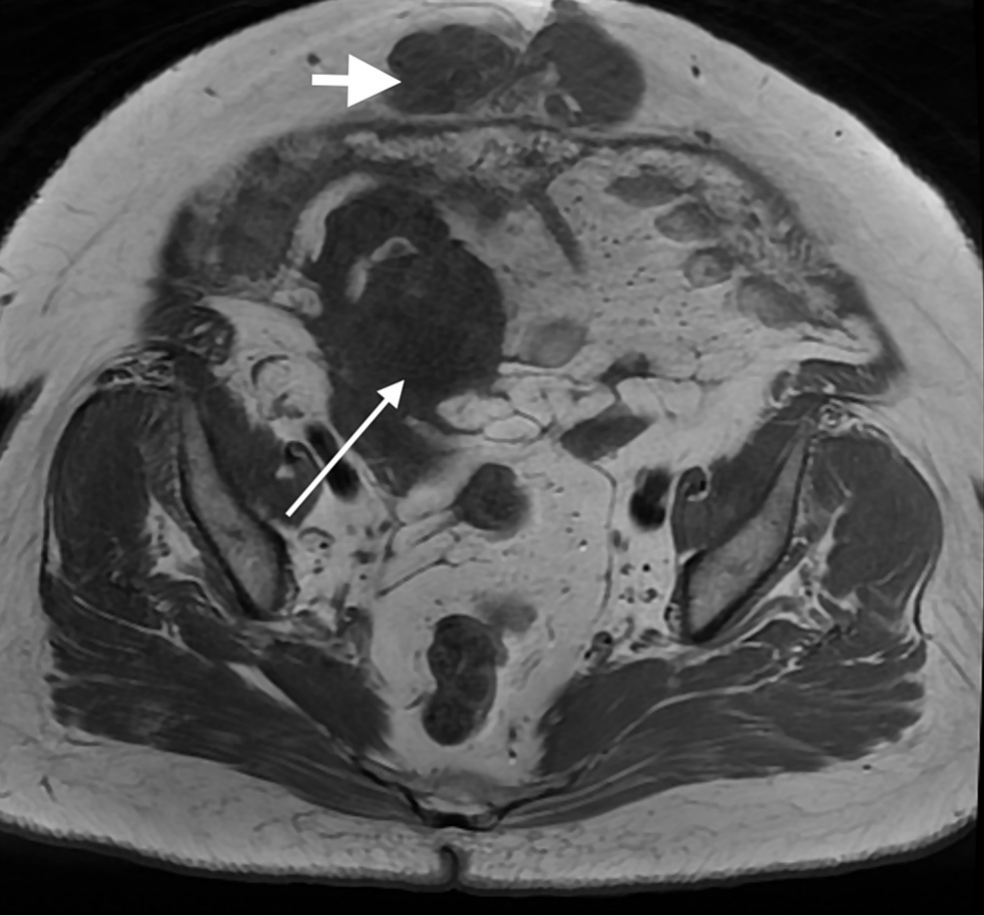
MRI of the abdomen and pelvis in an axial T1WI sequence. An irregularly-shaped low signal intensity structure is seen, corresponding to the lesions seen on the CT images (long arrow). Another low signal lesion is seen at the subcutaneous tissue of the mid abdomen representing the herniated component (short arrow).

**Figure 4:**
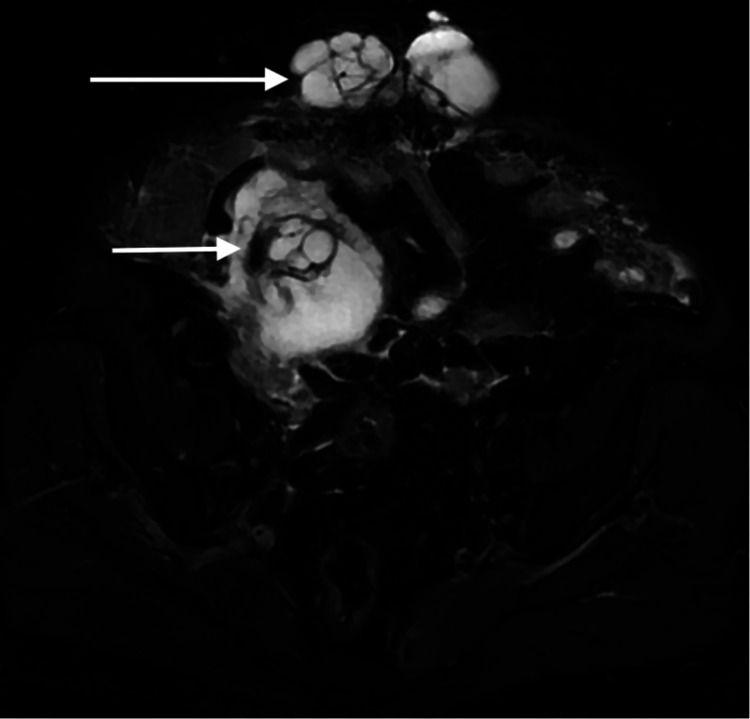
MRI of the abdomen and pelvis in an axial T2 fat sat sequence. On fat-suppressed T2WI MR of the lower abdomen, the lesion is of high signal intensity and shows multiple internal thin and smooth septations (short arrow). The mass is surrounded by high signal intensity fluid. The extension of the multi-septate lesion through the abdominal wall defect is also demonstrated in this image (long arrow).

**Figure 5:**
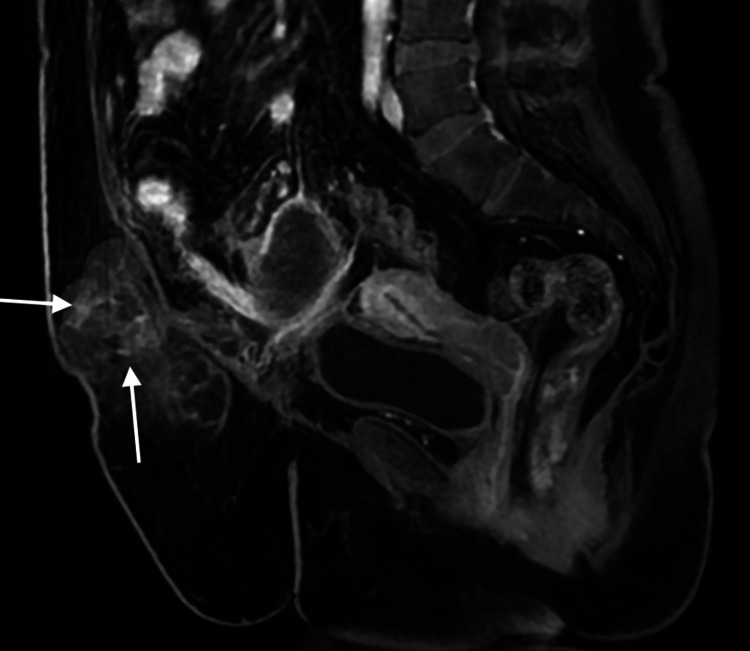
MRI of the abdomen and pelvis in a sagittal T1 fat sat sequence, post IV contrast administration. IV enhanced fat-suppressed T1WI shows enhancement of lesion, mostly at the internal septae (arrows). The craniocaudal dimensions of the hernial sac are better demonstrated in this sagittal sequence.

A 52-year-old woman with known history of diabetes presented to the surgical clinic complaining of a midline lump which she noticed recently. The patient also complained of diffuse abdominal bloating with discomfort that she had been feeling for three months. There was no history of nausea, vomiting, fever or chills. On examination, the patient was discovered to have an irreducible abdominal midline hernia. The patient was vitally stable with normal bowel motion and no signs of obstruction. The surgical team requested a CT scan with oral contrast for evaluation of the contents of the hernial sac. CT showed an irregular, predominantly low-density structure at the right adnexa with a focus of thin curvilinear peripheral calcification (Figure 1). This lesion appeared to extend anteriorly to the subcutaneous abdominal fat through a small defect in the abdominal wall (Figure 2). MRI of the abdomen and pelvis was ordered to further assess this lesion. On MRI, the origin of the lesion was better demonstrated as it was continuous with the cecal pole. There was complete loss of the fat plane between the lesion and the adjacent right adnexa, however, there was no definite sign to suggest the origin, arising from the right adnexa. The lesion showed an intermediate T1 and high T2 signal intensity indicating mucinous content (Figures 3, 4). In post-contrast images the lesion showed heterogeneous enhancement (Figure 5). After that, the patient had a colonoscopy which showed irregular mucosa around the appendicular orifice with exudates, and multiple biopsies were obtained. Biopsy only showed an area of active colitis with no dysplasia or evidence of malignancy. The patient then underwent an exploratory laparotomy which was abandoned as mucin material was diffusely spread within the abdominal cavity. No appendectomy or right hemicolectomy was performed. The surgeon did, however, obtain a specimen from the omentum that was sent for histopathology. The histopathology report indicated that the omental mass was consistent with peritoneal adenomucinosis, metastasizing from a primary low-grade appendiceal mucinous neoplasm. The patient recovered well from the procedure and was referred to a tertiary center for cytoreductive surgery and hyperthermic intraperitoneal chemotherapy (CRS-HIPEC).

## Discussion

MA is defined as chronic accumulation of mucin within an appendix, resulting in cystic dilatation of its lumen. The entity was first described by Rokitansky in 1842 and it is uncommon, with an incident rate of 0.2%-0.7% [1-2]. MA may occur secondary to luminal obstruction or due to mucus hypersecretion from an appendiceal cystadenoma/adenocarcinoma [4]. The majority of patients with mucoceles are asymptomatic until they present to the emergency department with symptoms mimicking acute appendicitis, e.g., right lower quadrant pain, nausea, and vomiting. Other cases may be discovered incidentally on imaging done for other purposes or during surgery for a different etiology [5]. Laboratory tests are non-specific and may show an elevated carcinoembryonic antigen (CEA) and cancer antigen (CA) 19-9 [6]. Histologically, four pathological entities are described with MA: 1) retention mucoceles secondary to outflow obstruction, 2) mucoceles with villous hyperplastic epithelium, 3) mucinous cystadenoma, 4) malignant cystadenocarcinoma [4]. 

Imaging features of non-complicated MA overlap with findings of acute appendicitis as both present as dilated appendices. However, the presence of cystic dilatation (> 1.4 cm) and luminal calcification can reliably differentiate between the two entities [7]. On MRI, the mucinous content of the lesion makes a MA more likely. A ruptured MA makes the imaging diagnosis more challenging as the lesion could be confused with an adnexal mass. However, its location is inseparable from the cecal pole making the adnexal origin unlikely. An ultrasound can be performed when a patient complains of right lower quadrant pain that mimics appendicitis. Ultrasound can show a hypoechoic structure with thin walls originating from the caecal pole, a hypoechoic structure with internal septations and internal echos, or a complex mass with posterior acoustic enhancement [8]. 

Complications of MA include ureteral obstruction and appendiceal torsion. MA can also serve as a leading point for intussusception [9]. Garg et al. described a case of intestinal obstruction occurring secondary to obstruction of the ileocecal valve by an MA [10]. Furthermore, mucocele can rupture which may result in PMP, a common and possibly lethal complication [9]. 

PMP is characterized by the presence of mucinous ascites within the peritoneal cavity. The condition occurs secondary to rupture of a mucinous tumor, commonly from the appendix or ovaries. Although benign, PMP exhibits a “borderline malignant” behavior [5]. On imaging, PMP may show septated ascites with immobile echos, suggestive of pseudomyxoma peritonei [10]. When a large amount of ascites is present CT would show a complex and hypodense cystic mass that characteristically scallops the liver and spleen and centrally displaces the bowel loops, with or without omental thickening [11]. 

Abdominal hernias, characterized by the protrusion of abdominal contents from the abdominal cavity, are a common asymptomatic finding. Patients may present with a reducible or irreducible hernia. When an irreducible hernia is encountered the differential diagnosis includes incarceration of the hernial sac [12]. In our case, the patient presented with an irreducible hernial sac due to the occupation of the sac by thick fluid. And so given the cystic nature of PMP, thick ascites may go through an existing hernial cavity, further complicating the clinical picture. Lee et al. described a case of ruptured mucocele that presented as an irreducible inguinal mass where thick ascites filled the hernial sac [13]. Ren et al. described a similar case where the patient presented as an umbilical hernia which, unlike our case, was reducible [14]. 

In females, the clinical presentation may be confusing for the physician. One study reports a case that was initially misdiagnosed on US and MRI as an adnexal mass until the final diagnosis was reached on pathology. This is thought to be related to the non-specific appearance on imaging, especially on transvaginal ultrasound [15]. Another study reports a case where a ruptured mucocele was misdiagnosed as a chronic tubo-ovarian abscess, based on the presence of a fluid-filled adnexal mass on ultrasound in the context of fever and tachycardia [16].

Appendiceal mucoceles are treated surgically. A laparoscopic appendectomy can be performed, with a risk of port side recurrence. Other operations that can be performed in cases of MA include right hemicolectomies, omentectomies and laparoscopic colectomies, based on the extent of the disease and the presence of complications. When the lesion is limited to the appendix a right hemicolectomy is favored [17]. Patients who have a confirmed diagnosis of PMP are recommended to be treated with CRS-HIPEC. When the disease is considered unresectable with high risk, a palliative debulking surgery is recommended with or without HIPEC [18]. 

## Conclusions

Although MA is a rare entity, it is important for physicians to consider this pathology in the differential diagnosis of non-specific abdominal pain, acute appendicitis and even pelvic masses. As discussed previously, this is due to the various clinical presentations it could present as. Even when dealing with a female patient who presents with a right adnexal lesion, the radiologist should be mindful of this entity and keep a high level of suspicion when encountering typical imaging findings of MA. These include low-density lesions with curvilinear calcification at the appendix on CT and thick ascites presenting as dense fluid with scalloping of the liver and spleen on all modalities. As in this case, searching for other origins of the lesion in the lower abdomen is the key to reaching the correct diagnosis. Radiological studies aid in the process of making a diagnosis and ruling out complications. However, histopathology is the most important tool for reaching the final diagnosis. Early diagnosis is paramount in cases of MA not only to avoid the risk of rupture but to also allow enough time for the treating physician to tailor the most suitable treatment for the patient.
